# Interpretable machine learning models for predicting short-term prognosis in AChR-Ab+ generalized myasthenia gravis using clinical features and systemic inflammation index

**DOI:** 10.3389/fneur.2024.1459555

**Published:** 2024-10-09

**Authors:** Yanan Xu, Qi Li, Meng Pan, Xiao Jia, Wenbin Wang, Qiqi Guo, Liqin Luan

**Affiliations:** ^1^Department of Neurology, Nanjing Jiangbei Hospital, Nanjing, China; ^2^Department of Neurology, The Affiliated Brain Hospital of Nanjing Medical University, Nanjing, China; ^3^Department of Neurology, The Affiliated Hospital of Xuzhou Medical University, Xuzhou, China

**Keywords:** short-term prognosis, generalized myasthenia gravis, systemic inflammation index, machine learning, prognosis

## Abstract

**Background:**

Myasthenia Gravis (MG) is an autoimmune disease that causes muscle weakness in 80% of patients, most of whom test positive for anti-acetylcholine receptor (AChR) antibodies (AChR-Abs). Predicting and improving treatment outcomes are necessary due to varying responses, ranging from complete relief to minimal improvement.

**Objective:**

Our study aims to develop and validate an interpretable machine learning (ML) model that integrates systemic inflammation indices with traditional clinical indicators. The goal is to predict the short-term prognosis (after 6 months of treatment) of AChR-Ab+ generalized myasthenia gravis (GMG) patients to guide personalized treatment strategies.

**Methods:**

We performed a retrospective analysis on 202 AChR-Ab+ GMG patients, dividing them into training and external validation cohorts. The primary outcome of this study was the Myasthenia Gravis Foundation of America (MGFA) post-intervention status assessed after 6 months of treatment initiation. Prognoses were classified as “unchanged or worse” for a poor outcome and “improved or better” for a good outcome. Accordingly, patients were categorized into “good outcome” or “poor outcome” groups. In the training cohort, we developed and internally validated various ML models using systemic inflammation indices, clinical indicators, or a combination of both. We then carried out external validation with the designated cohort. Additionally, we assessed the feature importance of our most effective model using the Shapley Additive Explanations (SHAP) method.

**Results:**

In our study of 202 patients, 28.7% (58 individuals) experienced poor outcomes after 6 months of standard therapy. We identified 11 significant predictors, encompassing both systemic inflammation indexes and clinical metrics. The extreme gradient boosting (XGBoost) model demonstrated the best performance, achieving an area under the receiver operating characteristic (ROC) curve (AUC) of 0.944. This was higher than that achieved by logistic regression (Logit) (AUC: 0.882), random forest (RF) (AUC: 0.917), support vector machines (SVM) (AUC: 0.872). Further refinement through SHAP analysis highlighted five critical determinants—two clinical indicators and three inflammation indexes—as crucial for assessing short-term prognosis in AChR-Ab+ GMG patients.

**Conclusion:**

Our analysis confirms that the XGBoost model, integrating clinical indicators with systemic inflammation indexes, effectively predicts short-term prognosis in AChR-Ab+ GMG patients. This approach enhances clinical decision-making and improves patient outcomes.

## Introduction

Myasthenia gravis (MG) is an autoimmune disorder marked by autoantibody disruptions at neuromuscular junctions, affecting ocular, bulbar, limb, respiratory, and axial muscles. Its clinical diversity allows categorization into subgroups based on symptoms, antibody specificity, and onset age (1). Approximately 80% of patients with MG develop generalized weakness (2), and among these, 85% test positive for anti-acetylcholine receptor (AChR) antibodies (3). These anti-AChR antibody-positive (AChR-Ab+) generalized myasthenia gravis (GMG) patients constitute the majority of MG cases and are central to trials exploring new immunotherapies. Treatment responses in MG vary significantly, from complete symptom relief to minimal improvement or even progression (4). Unfortunately, a notable portion of patients show suboptimal responses (5), highlighting the urgent need to predict poor treatment outcome to improve therapeutic strategies.

Previous studies have associated traditional clinical characteristics such as disease duration, quantitative myasthenia gravis (QMG) score, and gender with short-term outcome in AChR-Ab+ GMG patients (6, 7). However, these indicators fail to capture the full range of predictive data available. Given that inflammation is a central element in MG pathogenesis (8, 9), exploring systemic inflammation markers—such as the neutrophil to lymphocyte ratio (NLR), platelet to lymphocyte ratio (PLR), lymphocyte to monocyte ratio (LMR), and systemic immune-inflammation index (SII)—could be valuable (10). These markers are recognized as significant biomarkers in autoimmune diseases (11–13) and may illuminate the dynamics of AChR-Ab+ GMG. Nevertheless, the intricate and often nonlinear relationships between comprehensive medical data and clinical outcome create significant analytical challenges, diminishing the effectiveness of linear models such as logistic regression (Logit) for accurate predictions. In this context, the use of machine learning (ML)—a branch of artificial intelligence celebrated for its unmatched ability to unravel complex patterns in large and intricate datasets—is crucial for developing a robust predictive model (14). Common ML classifiers, including support vector machines (SVM) and extreme gradient boosting (XGBoost), have shown versatile applications in various fields such as oncology (15), cardiology (16), and MG (17). Despite this, research remains sparse on ML models that combine systemic inflammation indices with traditional clinical indicators to predict short-term prognosis in AChR-Ab+ GMG patients.

Although Liang et al. (6) and Zhao et al. (7) developed a predictive model for short-term prognosis in patients with AChR-Ab+ GMG, their work primarily focused on clinical characteristics with less emphasis on the systemic inflammation index. Furthermore, their reliance on traditional linear models instead of more advanced ML techniques compromised the precision of their predictions. In this context, our study aims to develop and validate an interpretable ML model that integrates systemic inflammation indices with traditional clinical indicators. Our goal is to predict the short-term prognosis of AChR-Ab+ GMG patients to guide personalized treatment strategies.

## Methods

### Ethics approval

This retrospective study adhered to the Declaration of Helsinki principles and was approved by the Ethics Committee of Nanjing Jiangbei Hospital (No. 2024062). Informed consent was obtained from all participants or their relatives.

### Patient selection

**Figure 1:**
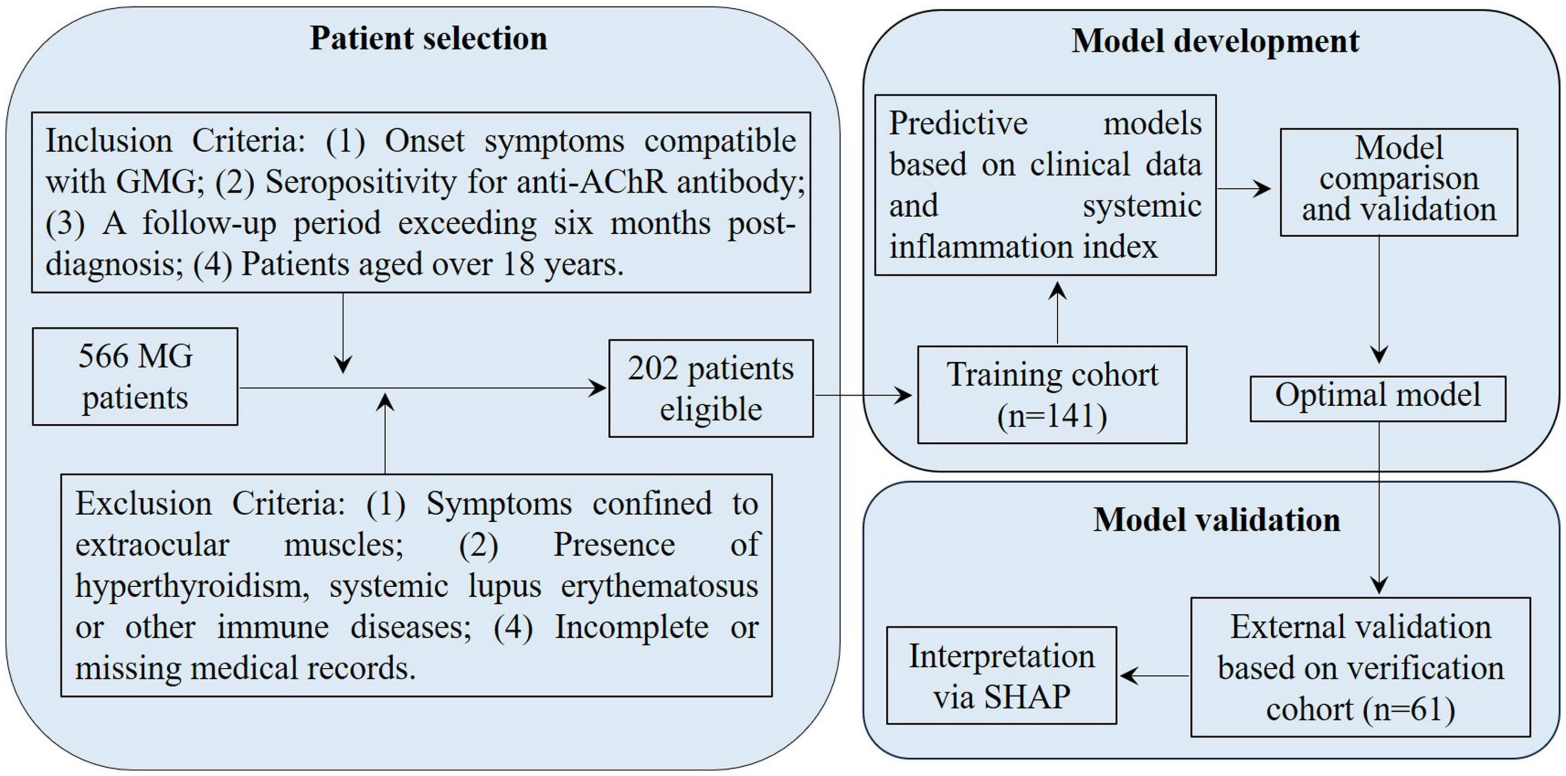
Flowchart for patient selection and cohort distribution in developing and validating predictive models for AChR-Ab+ GMG patients. GMG, generalized myasthenia gravis; AChR, acetylcholine receptor; Ab, antibody; MG, myasthenia gravis; SHAP, Shapley Additive Explanations.

From January 2016 to December 2023, 566 MG patients were screened at Nanjing Jiangbei Hospital, the Affiliated Brain Hospital of Nanjing Medical University, and the Affiliated Hospital of Xuzhou Medical University. The inclusion criteria for our study included onset symptoms compatible with GMG, seropositivity for anti-AChR antibody, a follow-up period exceeding 6 months post-diagnosis, and patients aged over 18 years. The exclusion criteria encompassed symptoms confined to extraocular muscles, the presence of hyperthyroidism, systemic lupus erythematosus, or other immune diseases, and incomplete or missing medical records. After thorough screening, 202 GMG patients were enrolled in our study. To prevent overfitting in the predictive model, 141 patients from January 2016 to May 2020 were involved in the training cohort and 61 patients from June 2020 to December 2023 were assigned to the external verification cohort (Figure 1). For treatment, 109 patients were administered prednisone acetate, and 143 received tacrolimus, with 50 of these also taking a combination of prednisone acetate tablets and tacrolimus capsules. All patients were prescribed pyridostigmine.

### Outcome measures

The primary outcome of this study was the Myasthenia Gravis Foundation of America (MGFA) post-intervention status assessed after 6 months of treatment. Prognoses were classified as “unchanged or worse” for poor outcome and “improved or better” for good outcome. This outcome measure, which is widely used in clinical and research settings, demonstrates the robustness and recognized utility of the MGFA post-intervention status in evaluating treatment effectiveness for MG (18).

### Data collection

To comprehensively evaluate treatment efficacy predictors in AChR-Ab+ GMG patients, we analyzed a range of clinical and systemic inflammation indicators. Clinical features assessed included age at onset, gender, body mass index (BMI), systolic and diastolic blood pressures (SBP and DBP), scores from the Myasthenia Gravis Foundation of America (MGFA), Quantitative Myasthenia Gravis (QMG), Myasthenia Gravis-Activity of Daily Living (MG-ADL), and the 15-item Myasthenia Gravis Quality of Life questionnaire (MG-QoL). We also considered thymectomy, thymoma presence, autoimmune disease comorbidity, disease duration, anti-AChR antibody titers, and hemoglobin (Hb) levels. Systemic inflammation was evaluated using white blood cell count (WBC), neutrophil, lymphocyte, platelet, monocyte, neutrophil-to-lymphocyte ratio (NLR), platelet-to-lymphocyte ratio (PLR), lymphocyte-to-monocyte ratio (LMR), and the systemic immune-inflammation index (SII), calculated using the formula (platelets * neutrophils)/lymphocytes. The disease duration was defined as the time from the onset of MG symptoms to the patient’s first hospital visit.

### Data preprocessing

Before developing the prediction model, we undertook a crucial data preprocessing phase to ensure the process’s fairness. This phase involved normalizing all data, covering both clinical features and systemic inflammation index. We applied *Z*-score normalization to continuous variables to standardize them to a mean of zero and a standard deviation of one. Categorical variables were converted to binary format, assigned values of “0” or “1.”

### Selection of features

To maintain a straightforward model, we applied student’s *t*-test, Mann–Whitney *U* test, and chi-square test to identify variables that significantly differed between the groups with good and poor outcomes. We then employed the least absolute shrinkage and selection operator (LASSO) regression with five-fold cross-validation for dimensionality reduction. Finally, variables with non-zero coefficients were analyzed using multivariable logistic regression to identify independent risk factors, thus constructing a ML model.

### Derivation and internal validation of ML models

To evaluate the short-term prognosis risk in AChR-Ab+ GMG patients, we utilized four established ML classifiers: Logit, random forest (RF), SVM, and XGBoost. Logit, a linear method, is essential for binary classification due to its straightforwardness and ease of interpretation, establishing it as a fundamental model (19). RF, developed from decision trees, is employed in classification models. It operates by each decision tree in the ensemble classifying the input data independently. Then, RF aggregates these predictions to determine the most common outcome. This method uses multiple decision tree models, leveraging varied data samples from the dataset to enhance prediction accuracy (20). SVM exemplifies kernel-based techniques due to its proficiency in identifying high-dimensional patterns. This robust classification algorithm focuses on establishing an efficient class-separating hyperplane, enhancing performance in complex datasets with numerous features (21). Finally, XGBoost, a tree-based gradient boosting algorithm that constructs an ensemble of weak decision trees to form a robust model, is known for its strong resistance to overfitting (22). It is notably flexible, managing diverse data types and formats without extensive feature engineering. Additionally, XGBoost excels in structured data problems, often surpassing other algorithms in predictive accuracy (23).

Our predictive models were based on clinical features, systemic inflammation index, and their combination, each forming a unique analytical base. During the training phase, to prevent overfitting, we implemented a triple-repeated five-fold cross-validation method. In this approach, each of the five iterations selects a unique fold as the internal testing set, while the remaining four folds serve as the internal training set. Additionally, this entire process is repeated three times to enhance accuracy further. The cumulative average of these three repetitions provides a reliable estimate of error rate (24). For the RF classifier, we configured 500 trees with node splitting based on the square root of the total features. In SVM, we selected a radial basis function (RBF) kernel, effectively handling non-linear data, and fine-tuned its hyperparameters—adjusting the cost parameter through a grid search of [0.1, 1, 10] and the gamma parameter at [0.001, 0.01, 0.1]. For XGBoost, we meticulously chose parameters to balance model complexity and accuracy, setting a learning rate of 0.02, a maximum tree depth of 4, and deploying an ensemble of 600 trees.

Following model development, each was subjected to comprehensive internal validation, evaluating its discrimination, calibration, and clinical utility. The optimal model was chosen based on its superior discrimination, strong calibration, and practical value in a clinical setting.

### External validation and interpretability of ML models

To ensure the robustness of our models, we performed external validation. This rigorous assessment confirmed their discriminative ability, calibration, and clinical applicability, providing a comprehensive view of their predictive capabilities. Additionally, after selecting the optimal predictive models, we explored the individual contributions of each variable using the Shapley Additive Explanation (SHAP) methodology. SHAP is based on the Shapley value from game theory, developed by economist Lloyd Shapley. This method and its extensions help in explaining machine learning model outputs through optimal credit distribution for local explanations (25). For example, Bi et al. (26) applied SHAP to measure the contribution of each feature in a model by calculating individual SHAP values for training samples. By aggregating these values, they ranked features according to their importance in predicting positive outcomes (26). SHAP’s interpretability is enhanced by visual plots where each point represents a sample, colored to denote the feature’s value—yellow for higher values and blue for lower ones, with the intensity of the color showing the magnitude of the feature value. We used the SHAP dependence plot to assess the significance of specific features and their effects on the model’s output. The SHAP force plot is designed to analyze and interpret the prediction outcomes for an individual sample.

### Statistical analysis

We employed a customized statistical approach tailored to the data type. We applied the chi-square test to categorical variables and used the Shapiro–Wilk test to evaluate the distribution of continuous variables. This assessment determined the appropriate use of either the Mann–Whitney *U* test or the independent-sample *t*-test for further analysis. To evaluate model performance, we employed receiver operating characteristic (ROC) curve analysis, which included metrics such as area under the curve (AUC), precision, recall, and F1 score to assess discrimination capability. DeLong’s test was used for AUC comparisons. Additionally, model fit was evaluated using calibration curve analysis and the Brier score to gauge the precision of probability predictions. Decision curve analysis (DCA) was conducted to estimate the net benefits of our models across various threshold probabilities, emphasizing their clinical value. Statistical analyses were carried out using IBM SPSS Statistics (version 22.0) and Python (version 3.7.1).

## Results

### Patient characteristics

The recruitment of study participants is illustrated in the flow diagram (Figure 1), with 202 out of 566 patients successfully enrolled. The poor outcome rates for AChR-Ab+ GMG patients after 6 months of standard therapy were similar between groups: 29.1% (41/141) in the training cohort and 27.9% (17/61) in the external validation cohort, with no statistically significant difference (*χ*^2^ = 0.030, *p* = 0.862). Data in Table 1 confirm these findings, showing consistent distributions of clinical features and systemic inflammation indices across both cohorts, with no significant disparities (all *p* > 0.05).

**Table 1:** Comparisons of clinical parameters and systemic inflammation markers between the training and external verification cohorts.

Variables	Training cohort (*N* = 141)	External verification cohort (*N* = 61)	*p*-value
Clinical parameters			
Age at onset, year, median (IQR)	61.00 (50.00, 74.00)	59.00 (49.00, 75.00)	0.853^c^
Gender, *n* (%)			0.775^b^
Female	81 (57.4)	33 (54.1)	
Male	60 (42.6)	28 (45.9)	
BMI, kg/m^2^, median (IQR)	24.10 (22.60, 32.30)	25.90 (22.90, 32.90)	0.759^c^
SBP, mm Hg, mean ± SD	122.88 ± 19.51	121.48 ± 18.58	0.634^a^
DBP, mm Hg, median (IQR)	79.00 (69.00, 88.00)	79.00 (69.00, 88.00)	0.940^c^
MGFA classification, *n* (%)			0.885^b^
II	85 (60.3)	39 (63.9)	
III	48 (34.0)	19 (31.1)	
IV	8 (5.7)	3 (4.9)	
QMG score, median (IQR)	10.00 (8.00, 13.00)	10.00 (8.00, 14.00)	0.632^c^
MG-ADL score, median (IQR)	6.00 (3.00, 9.00)	6.00 (2.00, 8.00)	0.485^c^
MG-QoL score, median (IQR)	15.00 (9.00, 22.00)	14.00 (8.00, 20.00)	0.343^c^
Thymectomy, *n* (%)			0.992^b^
No	115 (81.6)	49 (80.3)	
Yes	26 (18.4)	12 (19.7)	
Thymoma, *n* (%)			0.759^b^
No	104 (73.8)	43 (70.5)	
Yes	37 (26.2)	18 (29.5)	
Autoimmune disease			0.725^b^
No	120 (85.1)	50 (82.0)	
Yes	21 (14.9)	11 (18.0)	
Disease duration, month, median (IQR)	7.80 (5.00, 10.80)	6.90 (3.60, 10.40)	0.298^c^
Anti-AChR Abs titer, nmol/L, median (IQR)	8.00 (5.00, 11.00)	9.00 (7.00, 12.00)	0.405^c^
Pyridostigmine dosage, mg/day, median (IQR)	180.00 (180.00, 180.00)	180.00 (180.00, 180.00)	0.526^c^
Hb, g/L, median (IQR)	137.00 (128.00, 156.00)	144.00 (120.00, 158.00)	0.188^c^
Systemic inflammation markers			
WBC, 10^9^/L, median (IQR)	10.21 (6.99, 12.56)	10.00 (7.31, 12.36)	0.838^c^
Neutrophil, 10^9^/L, median (IQR)	6.58 (4.44, 9.69)	6.86 (5.26, 10.72)	0.453^c^
Lymphocyte, 10^9^/L, median (IQR)	2.43 (1.77, 2.90)	2.54 (2.06, 2.95)	0.311^c^
Platelet, 10^9^/L, median (IQR)	260.80 (205.06, 357.12)	264.00 (195.98, 371.74)	0.978^c^
Monocyte, 10^9^/L, median (IQR)	0.58 (0.42, 0.79)	0.61 (0.43, 0.87)	0.820^c^
NLR, median (IQR)	3.00 (2.00, 4.30)	3.10 (2.10, 4.20)	0.826^c^
PLR, median (IQR)	120.70 (93.30, 143.50)	105.90 (89.60, 140.70)	0.197^c^
LMR, median (IQR)	4.00 (2.80, 5.90)	4.30 (3.20, 5.50)	0.614^c^
SII, median (IQR)	767.75 (525.91, 1161.60)	772.98 (446.12, 1234.82)	0.920^c^

IQR, inter-quartile range; BMI, body mass index; SBP, systolic blood pressure; SD, standard deviation; DBP, diastolic blood pressure; MGFA, Myasthenia Gravis Foundation of America; QMG, quantitative myasthenia gravis; MG-ADL, myasthenia gravis-activity of daily living; MG-QoL, 15-item Myasthenia Gravis Quality of Life questionnaire; AChR, acetylcholine receptor; Abs, antibodies; Hb, hemoglobin; WBC, white blood cell; NLR, neutrophil-to-lymphocyte ratio; PLR, platelet-to-lymphocyte ratio; LMR, lymphocyte-to-monocyte ratio; SII, systemic immune-inflammation index.

a For independent sample *t*-test.

b For chi-square test.

c For Mann–Whitney *U*-test.

### Feature selection in the training cohort

**Figure 2:**
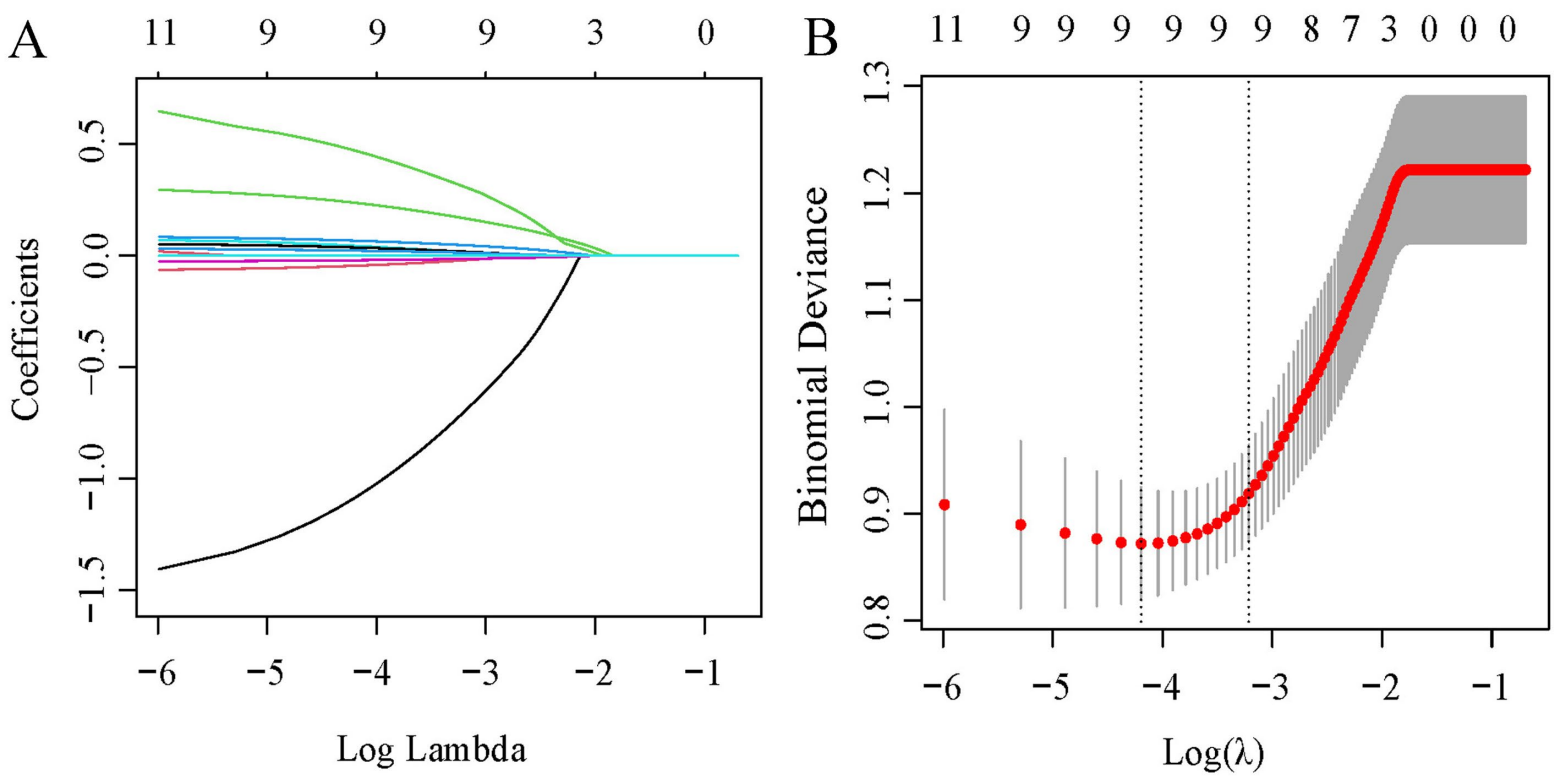
LASSO regression analysis for feature selection. **(A)** Coefficient profiles for 11 variables. **(B)** Determination of the optimal penalty coefficient lambda using five-fold cross-validation. The plot shows partial likelihood deviance against log (lambda), where lambda serves as the tuning parameter. Red dots represent average deviance values per model at each lambda, with error bars for standard error. Optimal values are marked with dotted vertical lines based on minimum criteria and the 1-SE rule.

**Figure 3:**
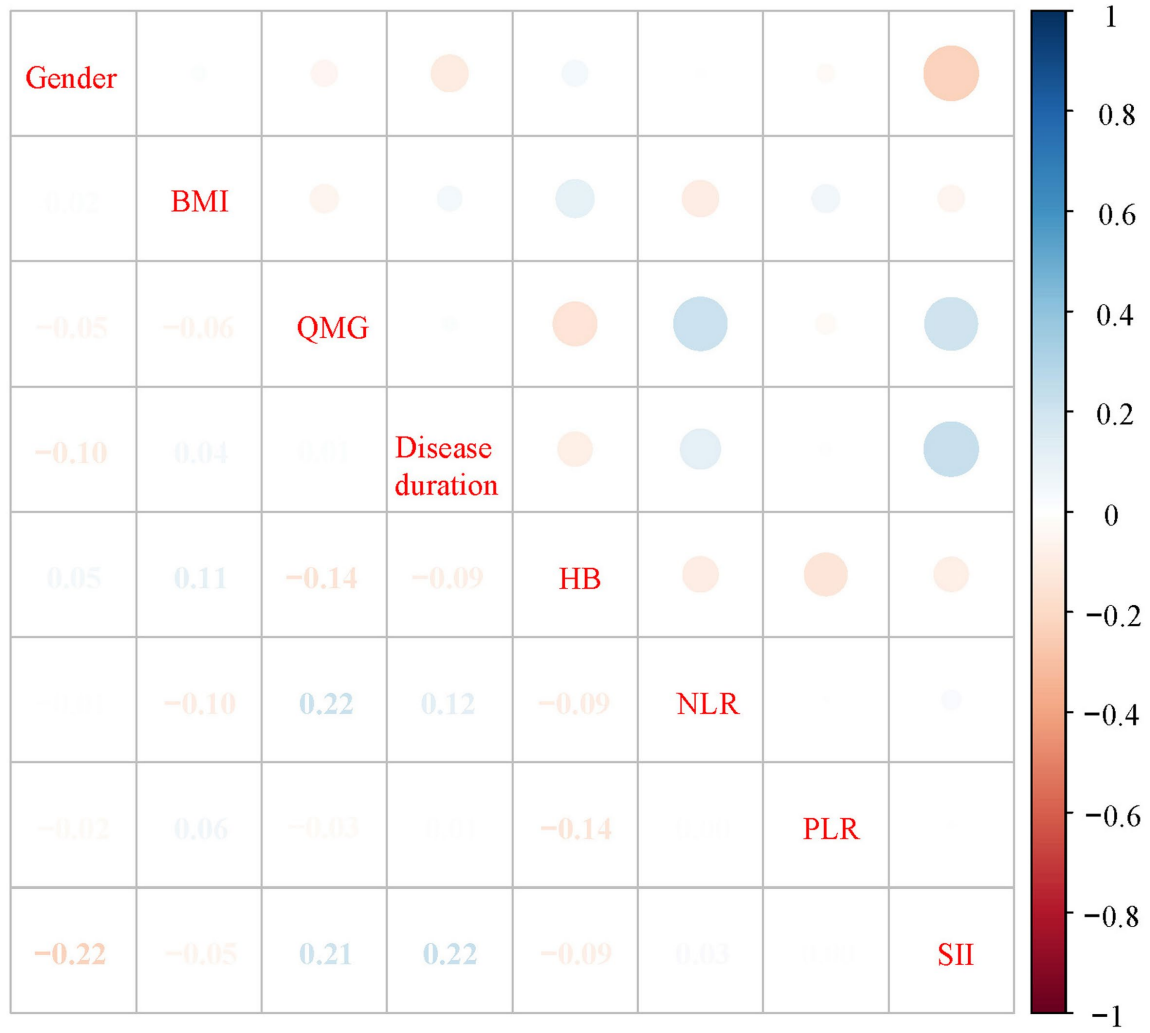
Correlation heatmap of variables.

Table 2 presents a comparison of clinical features and systemic inflammation index levels between patients with good and poor outcomes in the training cohort. The analysis reveals that poor outcome is associated with being female, having a lower BMI, higher QMG scores, longer disease durations, higher anti-AChR antibody titers, lower Hb levels, and elevated counts of WBCs, neutrophils, NLR, PLR, and SII—all with significant *p*-values (<0.05). We then applied LASSO regression and 10-fold cross-validation to refine the variable set, selecting nine variables using 1 standard error’s lambda (Figure 2): gender, BMI, QMG score, duration of disease before treatment, Hb, WBC, NLR, PLR, and SII. To further mitigate the impact of confounding factors, we conducted multivariate logistic regression on these variables to assess their roles as independent predictors of outcome in AChR-Ab+ GMG patients (Table 3). The analysis confirmed that all variables, except WBC, were significant independent predictors (all *p* < 0.05). The results of the correlation heatmap (Figure 3) indicate that all variable correlations are below 0.3, suggesting no significant correlations or multicollinearity among the variables. Finally, the ML model included the following variables: gender, BMI, QMG score, duration of disease before treatment, Hb, NLR, PLR, and SII. These key parameters underwent *Z*-score normalization to achieve a mean of zero and a standard deviation of one. This standardization streamlined their documentation and integration into the development of ML prediction models, thereby enhancing their predictive accuracy.

**Table 2:** Comparison of clinical parameters and systemic inflammation markers in patients with good and poor outcomes.

Variable	Good outcome (*n* = 100)	Poor outcome (*n* = 41)	*p*-value
Clinical parameters			
Age at onset, year, median (IQR)	59.00 (49.00, 74.00)	65.00 (52.00, 73.00)	0.529^c^
Gender, *n* (%)			0.003^b^
Female	49 (49.0)	32 (78.0)	
Male	51 (51.0)	9 (22.0)	
BMI, kg/m^2^, median (IQR)	26.55 (23.75, 32.73)	21.60 (20.70, 32.40)	0.048^c^
SBP, mm Hg, mean ± SD	123.07 ± 20.32	122.41 ± 17.58	0.857^a^
DBP, mm Hg, median (IQR)	79.00 (69.00, 88.00)	77.00 (68.00, 85.00)	0.706^c^
MGFA classification, *n* (%)			0.758^b^
II	62 (62.0)	23 (56.1)	
III	33 (33.0)	15 (36.6)	
IV	5 (5.0)	3 (7.3)	
QMG score, median (IQR)	8.00 (7.00, 13.00)	12.00 (10.00, 14.00)	<0.001^c^
MG-ADL score, median (IQR)	6.00 (3.00, 9.00)	4.00 (3.00, 8.00)	0.145^c^
MG-QoL score, median (IQR)	16.50 (9.00, 23.25)	15.00 (9.00, 18.00)	0.148^c^
Thymectomy, *n* (%)			0.354^b^
No	84 (84.0)	31 (75.6)	
Yes	16 (16.0)	10 (24.4)	
Thymoma, *n* (%)			0.463^b^
No	76 (76.0)	28 (68.3)	
Yes	24 (24.0)	13 (31.7)	
Autoimmune disease			0.212^b^
No	88 (88.0)	32 (78.0)	
Yes	12 (12.0)	9 (22.0)	
Disease duration, month, median (IQR)	6.85 (4.70, 8.83)	12.80 (7.30, 15.20)	<0.001^c^
Anti-AChR Abs titer, nmol/L, median (IQR)	7.00 (3.75, 10.00)	9.00 (5.00, 14.00)	0.023^c^
Pyridostigmine dosage, mg/day, median (IQR)	180.00 (180.00, 180.00)	180.00 (180.00, 180.00)	0.246^c^
Hb, g/L, median (IQR)	141.00 (133.75, 160.00)	115.00 (110.00, 157.00)	0.001^c^
Systemic inflammation markers			
WBC, 10^9^/L, median (IQR)	9.66 (7.00, 11.79)	11.69 (6.84, 17.24)	0.018^c^
Neutrophil, 10^9^/L, median (IQR)	6.05 (4.21, 8.82)	8.35 (5.46, 11.54)	0.010^c^
Lymphocyte, 10^9^/L, median (IQR)	2.54 (1.72, 2.94)	2.30 (1.85, 2.72)	0.191^c^
Platelet, 10^9^/L, median (IQR)	245.39 (197.39, 352.89)	280.37 (232.32, 375.65)	0.109^c^
Monocyte, 10^9^/L, median (IQR)	0.60 (0.44, 0.89)	0.54 (0.38, 0.72)	0.102^c^
NLR, median (IQR)	2.80 (1.98, 4.10)	4.30 (2.50, 5.50)	0.001^c^
PLR, median (IQR)	114.55 (89.35, 141.52)	137.00 (102.70, 155.80)	0.004^c^
LMR, median (IQR)	4.00 (2.77, 5.50)	4.10 (2.90, 6.10)	0.138^c^
SII, median (IQR)	666.48 (468.51, 987.05)	1206.57 (729.81, 1524.60)	<0.001^c^

IQR, inter-quartile range; BMI, body mass index; SBP, systolic blood pressure; SD, standard deviation; DBP, diastolic blood pressure; MGFA, Myasthenia Gravis Foundation of America; QMG, quantitative myasthenia gravis; MG-ADL, myasthenia gravis-activity of daily living; MG-QoL, 15-item Myasthenia Gravis Quality of Life questionnaire; AChR, acetylcholine receptor; Abs, antibodies; Hb, hemoglobin; WBC, white blood cell; NLR, neutrophil-to-lymphocyte ratio; PLR, platelet-to-lymphocyte ratio; LMR, lymphocyte-to-monocyte ratio; SII, systemic immune-inflammation index.

a For independent sample *t*-test.

b For chi-square test.

c For Mann–Whitney *U*-test.

**Table 3:** Validation of variables in LASSO regression using multivariable logistic analysis.

Variables	LASSO regression		Multivariable logistics regression	
	Coefficients	Lambda.1se	OR (95% CI)	*p*-value
Clinical parameters				
Gender	−0.6244961	0.0477387	0.209 (0.060–0.637)	0.009
BMI	−0.0149277		0.932 (0.656–0.998)	0.036
QMG score	0.15498217		1.397 (1.147–1.758)	0.002
Disease duration	0.04346586		1.104 (1.031–1.198)	0.009
Hb	−0.0126869		0.975 (0.949–0.999)	0.048
Systemic inflammation markers				
WBC	0.01484227		1.055 (0.974–1.151)	0.196
NLR	0.28073891		2.161 (1.206–4.141)	0.013
PLR	0.01192355		1.038 (1.015–1.065)	0.002
SII	0.0117102		1.999 (1.098–2.001)	0.003

Lambda.1se, among all lambda values, the lambda value of the simplest model within a variance of the mean value of the minimum target parameter is obtained; LASSO, least absolute shrinkage and selection operator; OR, odds ratio; CI; confidence interval; BMI, body mass index; QMG, quantitative myasthenia gravis; Hb, hemoglobin; WBC, white blood cell; NLR, neutrophil-to-lymphocyte ratio; PLR, platelet-to-lymphocyte ratio; SII, systemic immune-inflammation index.

### Comparing models for predicting poor outcome risk

**Figure 4:**
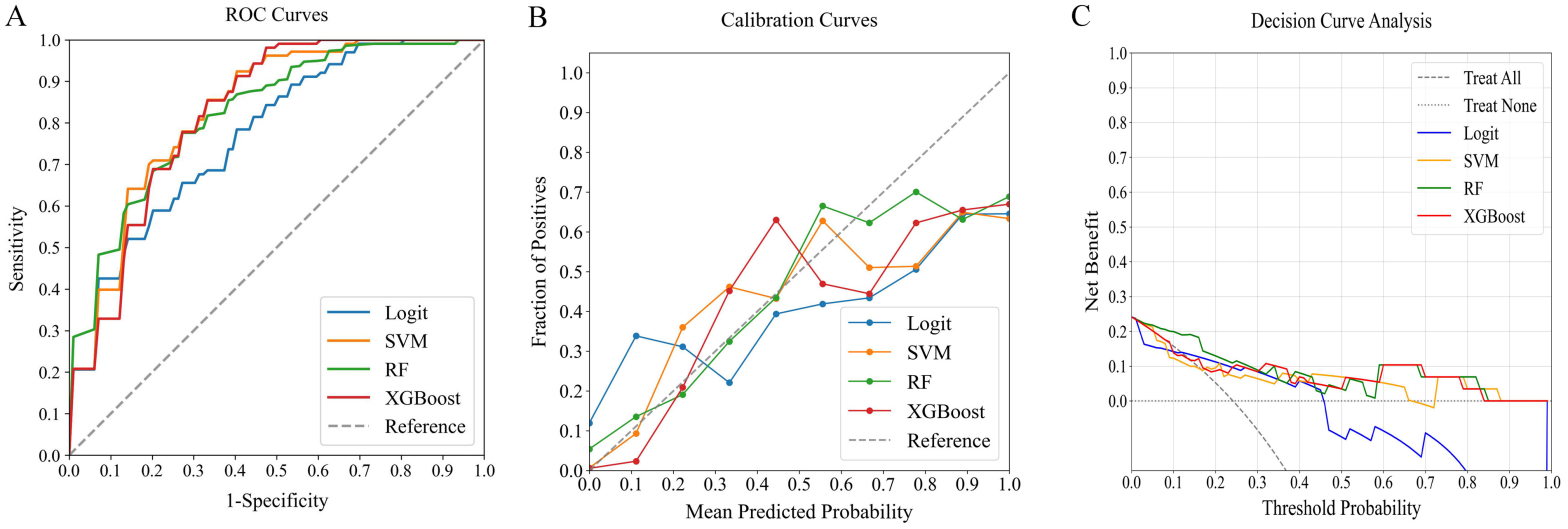
Comparative performance of ML classifiers (Logit, SVM, RF, XGBoost) on clinical data: **(A)** ROC curves, **(B)** calibration plots, and **(C)** DCA. They achieved ROC-AUCs of 0.772, 0.831, 0.819, and 0.824, respectively. ML, machine learning; ROC, receiver operating characteristic; AUC, area under the curve; DCA, decision curve analysis; Logit, logistic regression; SVM, support vector machine; RF, random forest; XGBoost, extreme gradient boosting.

**Figure 5:**
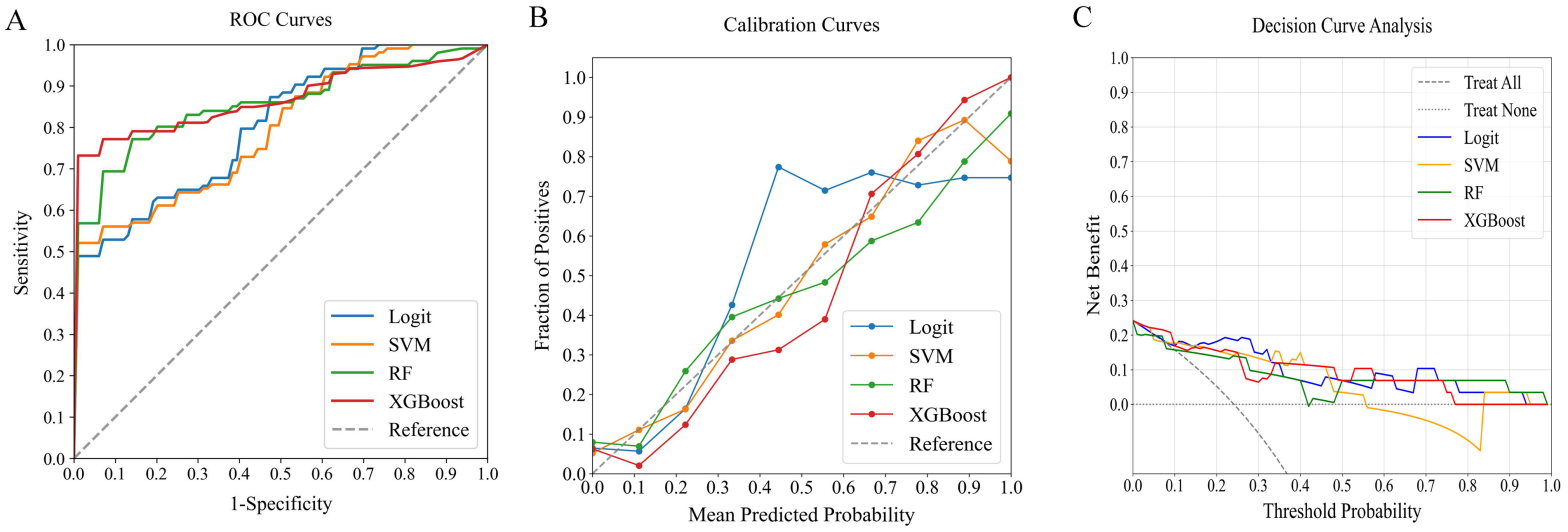
Comparative performance of ML classifiers (Logit, SVM, RF, XGBoost) on systemic inflammation index: **(A)** ROC curves, **(B)** calibration plots, and **(C)** DCA. They achieved ROC-AUCs of 0.804, 0.792, 0.855, and 0.854, respectively. ML, machine learning; ROC, receiver operating characteristic; AUC, area under the curve; DCA, decision curve analysis; Logit, logistic regression; SVM, support vector machine; RF, random forest; XGBoost, extreme gradient boosting.

**Figure 6:**
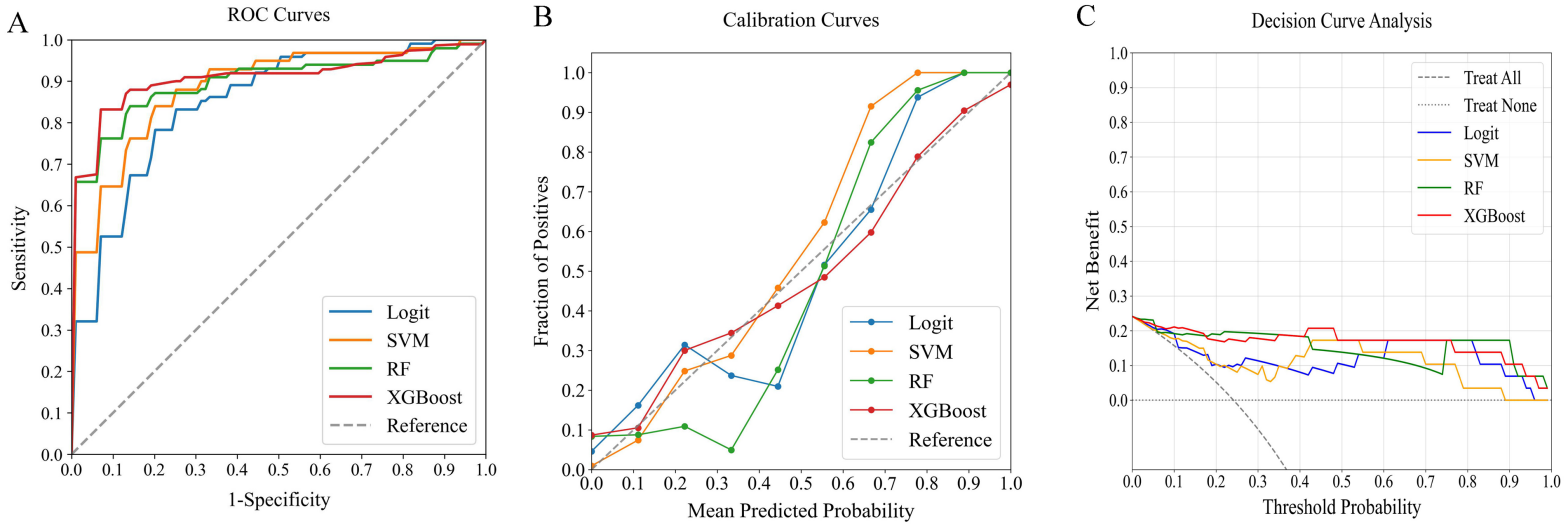
Comparative performance of ML classifiers (Logit, SVM, RF, XGBoost) on combined clinical data and systemic inflammation index: **(A)** ROC curves, **(B)** calibration plots, and **(C)** DCA. They achieved ROC-AUCs of 0.882, 0.872, 0.917, and 0.944, respectively. ML, machine learning; ROC, receiver operating characteristic; AUC, area under the curve; DCA, decision curve analysis; Logit, logistic regression; SVM, support vector machine; RF, random forest; XGBoost, extreme gradient boosting.

In our comprehensive analysis of predictive models for poor outcomes in AChR-Ab+ GMG patients, we assessed four ML classifiers: Logit, SVM, RF, and XGBoost. These classifiers were tested against three sets of predictors: clinical indicators, systemic inflammation indices, and their combination. Table 4 details the performance comparison of these models, while Figures 4–6 display the ROC curves, calibration plots, and DCA. Our findings indicate that models integrating both sets of predictors achieved better discriminative ability (AUC: 0.872–0.944) compared to those using solely clinical indicators (AUC: 0.772–0.831) or systemic inflammation measures (AUC: 0.792–0.855), with statistical significance confirmed by DeLong’s test (*p* < 0.05).

**Table 4:** Performance of ML classifiers in predicting poor outcome risk in AChR-Ab+ GMG using clinical data, systemic inflammation markers, and combined datasets.

Data type	ML classifier	AUC	Precision	Recall	F1 score	Brier score
Clinical parameters	Logit	0.772	0.602	0.432	0.503	0.042
	SVM	0.831	0.702	0.503	0.624	0.033
	RF	0.819	0.671	0.591	0.626	0.019
	XGBoost	0.824	0.582	0.633	0.732	0.025
Systemic inflammation markers	Logit	0.804	0.725	0.723	0.725	0.028
	SVM	0.792	0.641	0.714	0.799	0.009
	RF	0.855	0.835	0.726	0.772	0.010
	XGBoost	0.854	0.836	0.816	0.771	0.011
Combined clinical parameters and systemic inflammation markers	Logit	0.882	0.859	0.826	0.842	0.009
	SVM	0.872	0.801	0.823	0.842	0.010
	RF	0.917	0.912	0.821	0.899	0.015
	XGBoost	0.944	0.925	0.861	0.927	0.003

ML, machine learning; AChR-Ab+, acetylcholine receptor antibody-positive; GMG, generalized myasthenia gravis; AUC, area under the curve; Logit, logistic regression; SVM, support vector machine; RF, random forest; XGBoost, extreme gradient boosting.

Among the models that combined clinical indicators and systemic inflammation indices, XGBoost emerged as the most effective, achieving the highest AUC of 0.944 with superior calibration, especially above the 75% threshold. Performance was uniformly validated across all models in DCA. The performance of XGBoost was consistently strong across all key metrics, including precision, recall, F1 score, and Brier score. These results establish XGBoost as the optimal model for predicting poor outcome risk in AChR-Ab+ GMG patients.

### Assessing ML model using an external verification cohort

**Figure 7:**
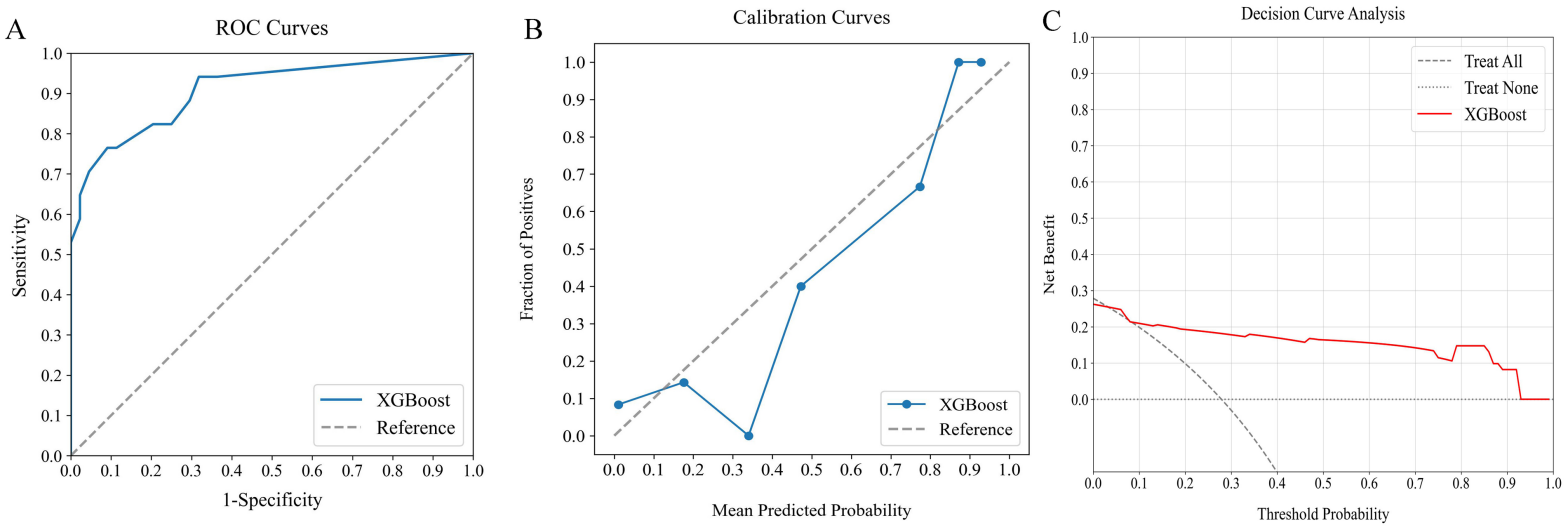
Assessing the predictive performance of the optimal ML model using an external verification cohort: **(A)** ROC curve (AUC = 0.908), **(B)** calibration curve, and **(C)** DCA. ML, machine learning; ROC, receiver operating characteristic; AUC, area under the curve; DCA, decision curve analysis.

The external verification cohort was utilized to evaluate the predictive accuracy of the XGBoost model for poor outcome, employing ROC, calibration, and DCA analyses (Figure 7). Although there was a slight decrease in performance relative to the training cohort, the XGBoost model maintained a strong discriminative ability, with an AUC of 0.908 (Figure 7A). The calibration curve demonstrated high agreement between the predicted risks and observed outcome (Figure 7B). Furthermore, the DCA curve confirmed the model’s effectiveness by showing significant net benefits (Figure 7C). These findings underscore the XGBoost model’s robustness and clinical value as a predictive tool for assessing poor outcome risk.

### Interpretation of the model

**Figure 8:**
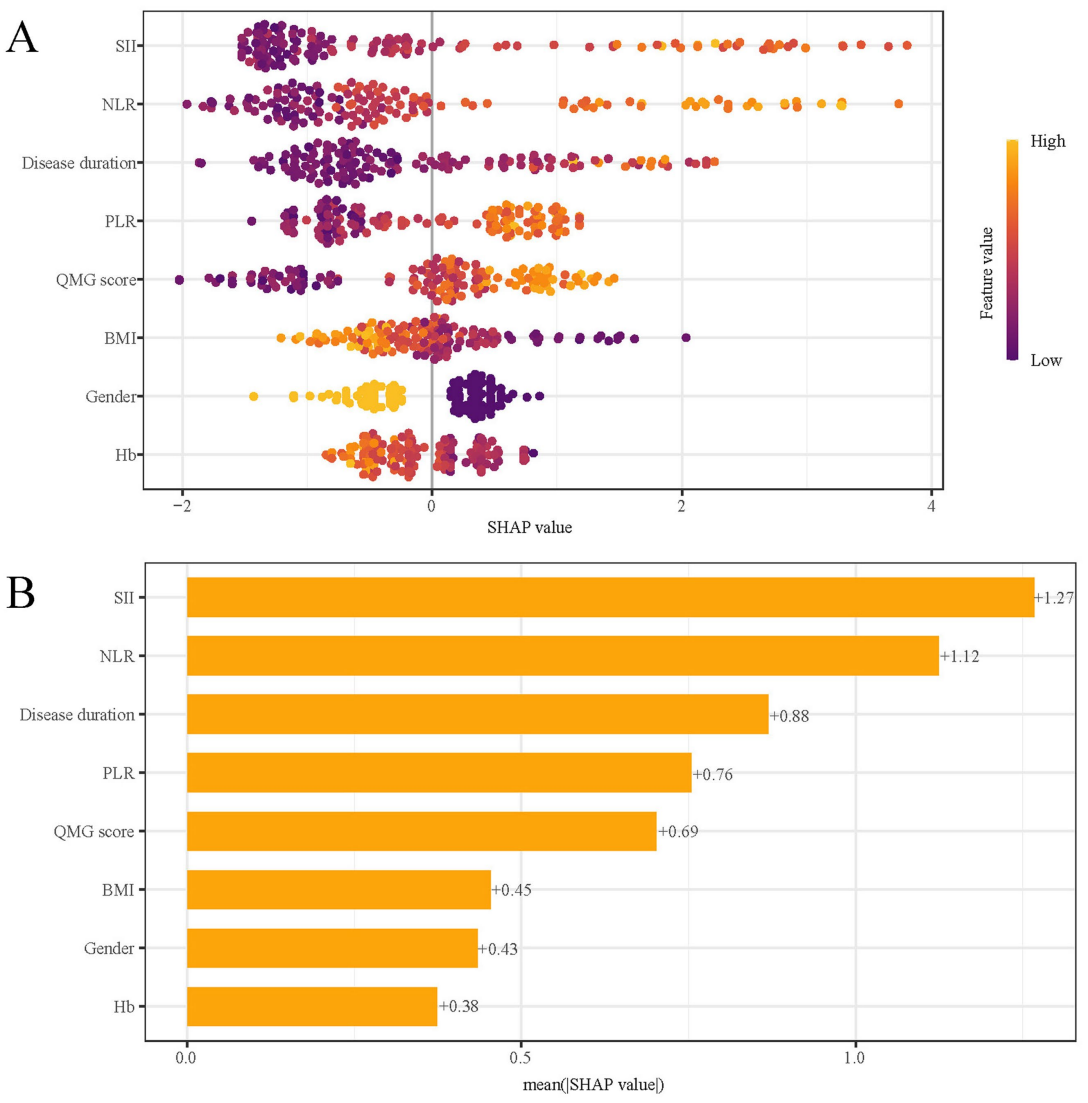
SHAP analysis of XGBoost model for predicting short-term prognosis. **(A)** Summary plot and **(B)** feature importance ranking. SHAP, Shapley Additive Explanations; XGBoost, extreme gradient boosting; SII, systemic immune-inflammation index; QMG, quantitative myasthenia gravis; NLR, neutrophil to lymphocyte ratio; PLR, platelet to lymphocyte ratio; BMI, body mass index; Hb, hemoglobin.

SHAP analysis was employed to elucidate the impact of individual features in the XGBoost model, quantifying the influence of each by calculating their absolute mean SHAP values. This approach ranked features by importance, revealing two clinical indicators and three systemic inflammation indices as the top five contributors (Figure 8). The SHAP summary plot (Figure 8A) is derived from estimates, allocating a specific data point to each feature for every patient. In this visualization, yellow signifies higher values and blue denotes lower ones. The SHAP values are displayed along the horizontal axis, where larger shapes highlight features with greater importance in forecasting the short-term prognosis of AChR-Ab+ GMG patients. The importance bar chart (Figure 8B) outlines the impact of each variable on prognosis prediction. In summary, ranked by decreasing significance, the key features are: SII, NLR, disease duration, PLR, QMG score, BMI, gender, Hb.

**Figure 9:**
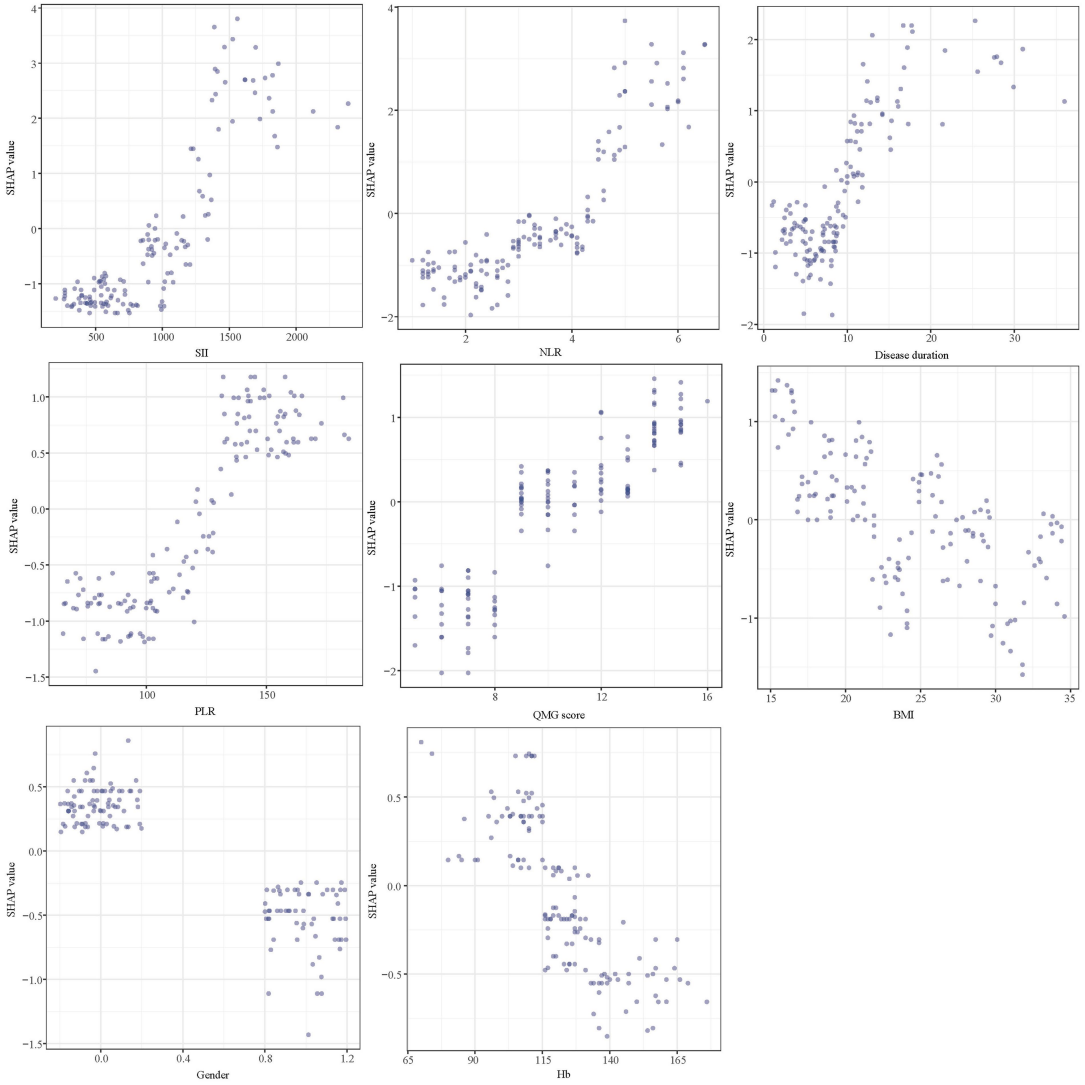
SHAP dependency plot of the XGboost model. SHAP, Shapley Additive Explanations; XGBoost, extreme gradient boosting; SII, systemic immune-inflammation index; NLR, neutrophil to lymphocyte ratio; PLR, platelet to lymphocyte ratio; QMG, quantitative myasthenia gravis; BMI, body mass index; Hb, hemoglobin.

Figure 9 presents SHAP dependence plots for each of the eight factors, elucidating their influence on the outcomes of the XGBoost model. Positive SHAP values indicate a higher risk of poor outcomes in AChR-Ab+ GMG patients. Our findings associate poor outcomes with several factors: increased SII, NLR, and PLR; longer disease duration prior to treatment; elevated QMG scores; female gender; lower BMI; and decreased Hb levels.

**Figure 10:**
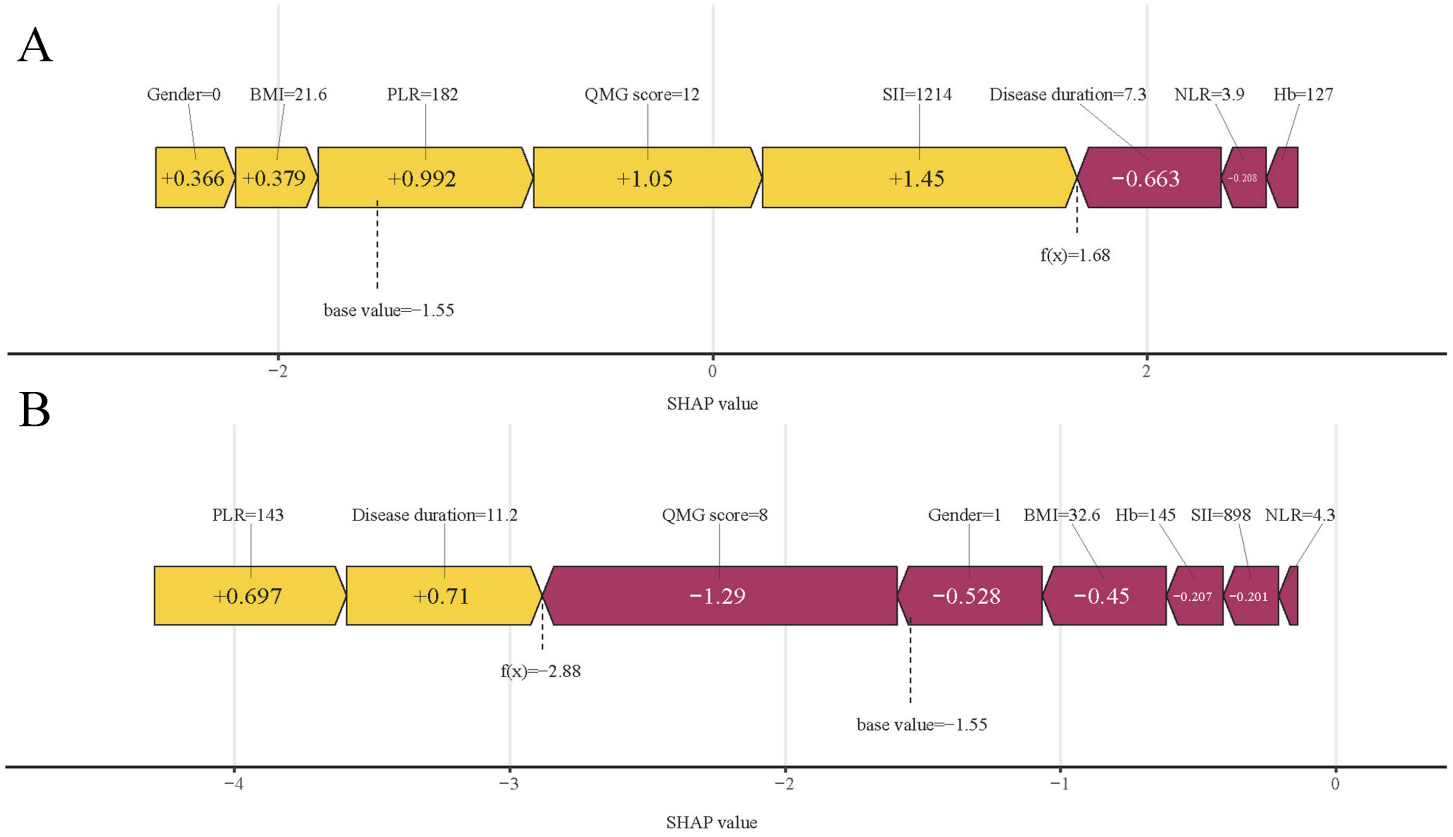
SHAP force plots illustrating individual prediction results: **(A)** for a patient with a poor outcome; **(B)** for a patient with a good outcome. SHAP, Shapley Additive Explanations; SII, systemic immune-inflammation index; QMG, quantitative myasthenia gravis; NLR, neutrophil to lymphocyte ratio; PLR, platelet to lymphocyte ratio; BMI, body mass index; Hb, hemoglobin.

In predictive modeling, the SHAP force plot clearly demonstrates how certain features affect individual patient outcomes (Figure 10). Yellow areas show features increasing the likelihood of poor outcomes in AChR-Ab+ GMG patients, while red areas show features decreasing this likelihood. The wider the color region, the more significant the impact. The value *f*(*x*) sums up the SHAP values for each patient, with the base value representing the average SHAP value across all patients. The upper panel illustrates an accurate prediction of a poor outcome, attributed to factors such as female gender and higher SII values (Figure 10A). The lower panel, in contrast, accurately identifies a patient likely to experience a good outcome, based on a lower QMG score and male gender, and others (Figure 10B). Using XGBoost, this approach effectively differentiates between patients at risk for poor or good outcomes, providing customized risk assessments.

## Discussion

MG is an autoimmune disease that affects various muscles, leading to generalized weakness in 80% of patients, most of whom test positive for anti-AChR-Abs (2, 3). Inflammation is key in MG, enhancing inflammatory factors, activating B cells, and producing autoantibodies (8, 9, 27). Due to varied treatment responses, precise predictive models are essential. Current models fail in accuracy as they overlook systemic inflammation and use traditional linear approaches instead of advanced ML techniques (6, 7). To address this deficiency, our study developed predictive models using four ML classifiers, incorporating clinical features, the systemic inflammation index, or a combination of both. Our analysis identified the XGBoost model, integrating both clinical features and the systemic inflammation index, as the most effective for predicting short-term prognosis. Notably, integrating SHAP analysis enhanced the interpretability of the XGBoost model, clarifying the influence of the systemic inflammation index in prognosis. This research signifies a substantial advancement in using ML to integrate clinical indicators with the systemic inflammation index for accurate short-term prognosis assessments. Early prediction of treatment response facilitates tailored treatment strategies, potentially offering more intensive or alternative therapies to high-risk patients. Such strategies are likely to increase therapeutic success, slow disease progression, reduce hospital stays, and enhance patient quality of life.

In our study, we selected ML models due to their proficiency in analyzing complex non-linear relationships between variables and outcomes, a capability that exceeds that of conventional linear models (28). We utilized four ML models to assess clinical indicators, the systemic inflammation index, and their integration. The models that combined both data types demonstrated superior efficacy in predicting short-term prognosis, likely because they capture a broader array of factors that directly influence outcomes. This comprehensive methodology significantly improved predictive accuracy.

Among the ML models we evaluated, XGBoost proved to be the most effective. It utilized clinical indicators and the systemic inflammation index to deliver high accuracy, a performance that was consistent even during external validation. Indeed, previous studies have developed ML techniques to predict short-term clinical outcome in MG patients. For example, Zhong et al. (17) analyzed clinical and other characteristics of MG patients with diverse antibody types using an ML model to predict their short-term outcomes. Our research specifically targets AChR-Ab+ GMG patients, with a focus on evaluating the systemic inflammation index to enhance prediction accuracy. To enhance the interpretability of this complex ML model, we employed SHAP analysis. The SHAP feature importance map visually represents the impact of each feature on a model’s output. It displays the SHAP values for each feature, indicating their range and the positive or negative effect on the model. High SHAP values correlate with significant influence (29). Each point in the plot corresponds to a sample, with bar graphs showing SHAP value distributions. The color of these bars reflects feature values within the sample. The position of each bar graph on the plot reveals the feature’s influence direction: leftward shifts indicate negative impacts, while rightward shifts suggest positive effects. This tool aids in identifying critical features for optimizing model performance and guiding feature selection (30, 31). The five most important predictors of short-term prognosis on the SHAP feature importance map include two clinical indicators and three related to the systemic inflammation index. Inflammatory mediators such as interleukins, interferons, and chemokines from inflammatory cells play a key role in modulating the immune response. Studies indicate that macrophages and monocytes in MG release cytokines, initiating inflammatory cascades that activate the immune system (32, 33). Supporting evidence includes detection of neuromuscular antigens like AChRs, germinal centers, elevated Tfh cell counts in the thymus, changes in microRNAs, and specific IFN signaling in thymic epithelial cell subpopulations in MG patients with thymoma (34, 35). The detection of circulating inflammatory indexes is easy to conduct and cost-effective. Based on the information above, we specifically focused on the circulating inflammatory markers in our study, predicting the short-term prognosis of AChR-Ab+ GMG patients, a focus that is rare in previous study. In our study, we observed that elevated levels of three key systemic inflammation indices—NLR, PLR, and SII—correlated with poor outcomes in patients with AChR-Ab+ GMG. The robustness of these indices against physiological, pathological, and physical variations makes them more effective than individual metrics such as neutrophils, lymphocytes, monocytes, or platelets (36, 37). Among these, the SII particularly stands out as it encapsulates the dynamic interplay and potential synergy among platelets, neutrophils, and lymphocytes (38). Consequently, compared to other markers like NLR and PLR, the SII potentially offers a more objective representation of the interactions between inflammatory and immune responses. Moreover, our model indicates that higher QMG scores and prolonged disease duration are associated with poorer treatment responses, aligning with the established correlation between these factors and increased disease severity and chronicity. This finding is consistent with prior research (6, 7), underscoring the importance of early and aggressive intervention in patients with severe symptoms or a lengthy disease history to enhance treatment outcomes. Using SHAP, XGBoost offered clear insights into how different factors affect outcomes, proving crucial for screening risks of poor outcomes. Integrating ML into this screening process holds promise for enabling clinicians to initiate early interventions that improve outcomes for AChR-Ab+ GMG patients.

This ML model could transform management practices in several ways according to our study. First, for patients with a higher systemic inflammation index, the model improves patient-physician communication by alerting about potential poor outcomes, which also allows clinicians to better prepare and proactively manage care. Second, it aids early-career clinicians by facilitating referrals for patients predicted to have poor outcomes to more specialized and experienced clinicians, thus reducing the risks associated with inexperience. Finally, other clinicians can input clinical features and systemic inflammation indices into our XGBoost ML models to obtain precise clinical predictions. The model also provides a SHAP force plot that illustrates the impact of each variable on the outcomes, enhancing diagnostic accuracy and understanding.

Our study yielded promising results, yet two limitations should be noted. Initially, it was limited to three institutions in the same region and involved only 202 patients, possibly reflecting regional biases and the constraints of a modest sample size, which may affect the generalizability of the findings. Furthermore, the exclusion of patients without comprehensive clinical records and the retrospective design of the study may further contribute to selection bias. Despite these issues, our research highlights the capability of ML models that integrate clinical indicators and the systemic inflammation index to predict the short-term prognosis of AChR-Ab+ GMG patients. Future research should adopt larger-scale, multi-center, prospective studies to enhance the model’s reliability and extend its applicability.

In conclusion, the XGBoost model excels in predicting the short-term prognosis of AChR-Ab+ GMG patients by integrating clinical indicators with the systemic inflammation index. This ML model enables precise risk assessment, aiding clinicians in informed decision-making and improving patient outcomes.

## Data Availability

The original contributions presented in the study are included in the article/supplementary material, further inquiries can be directed to the corresponding author.
